# Supplementary figures and images for: sc2MeNetDrug: A computational tool to uncover inter-cell signaling targets and identify relevant drugs based on single cell RNA-seq data
Source: PLoS Comput Biol. 2024 Jan 5;20(1):e1011785. doi: 10.1371/journal.pcbi.1011785 (PMC10796047; doi:10.1371/journal.pcbi.1011785)

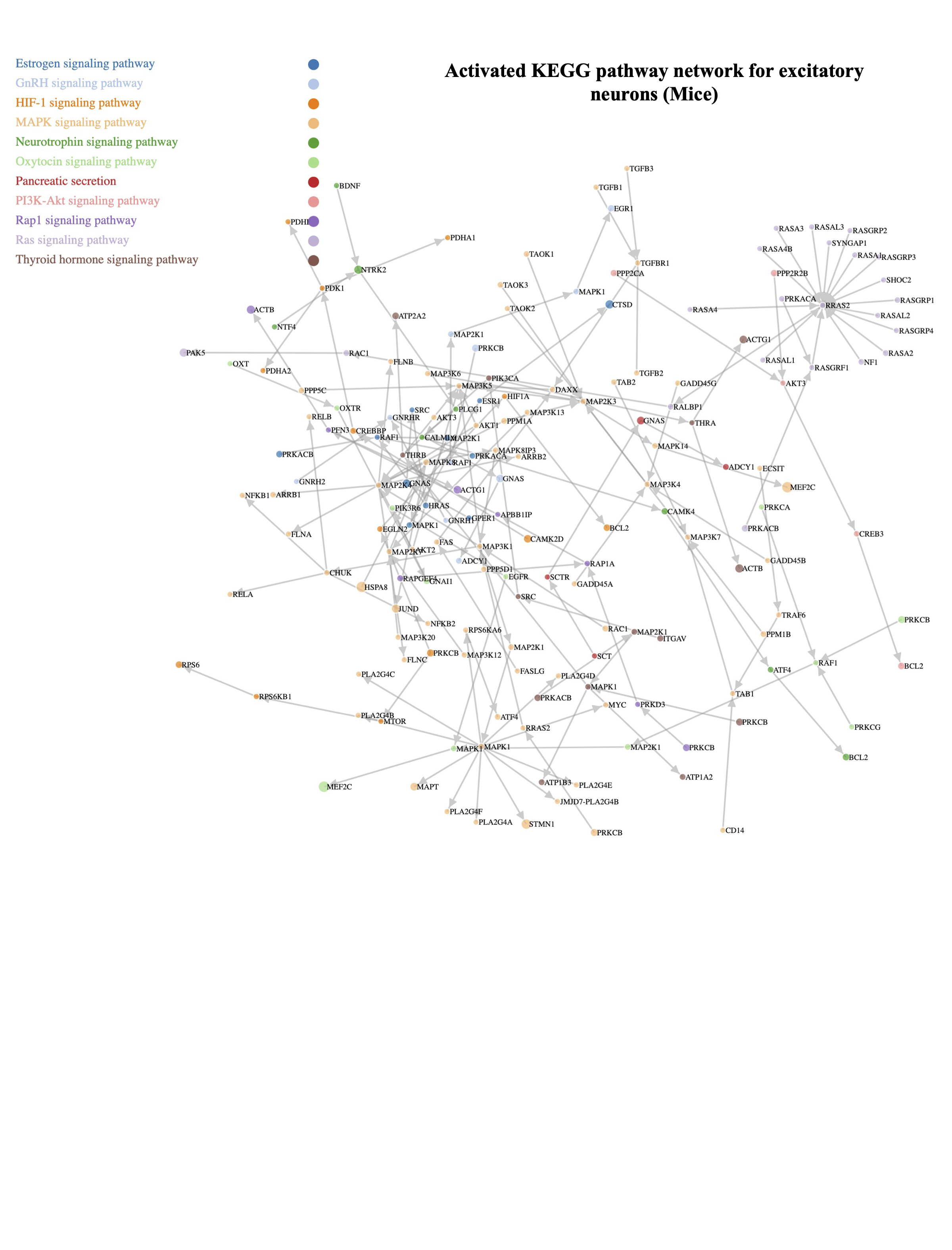

Supplement: S1 Fig — (TIFF) [file pcbi.1011785.s001.tiff]

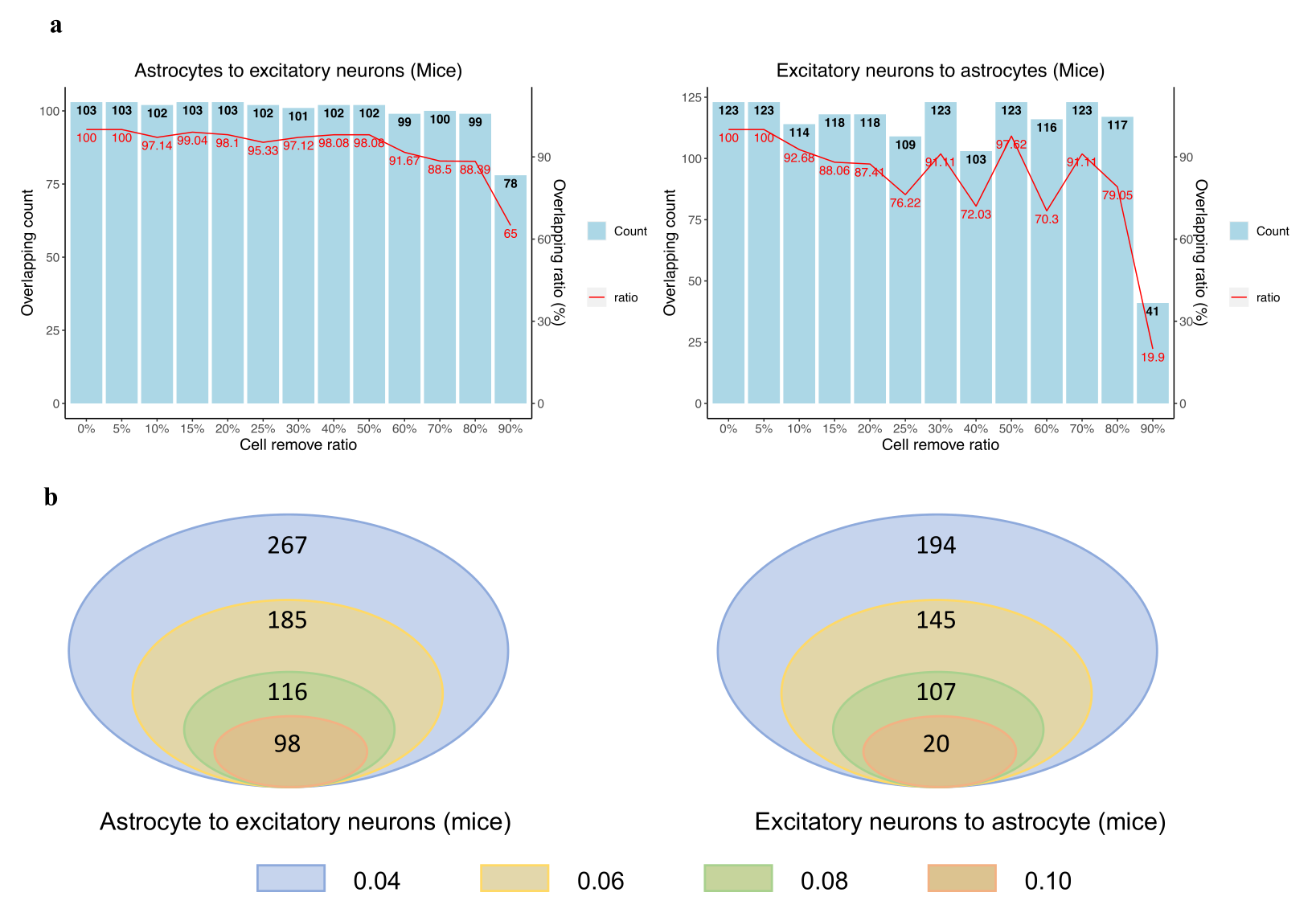

Supplement: S2 Fig — (a) Cell type sub-population effects. Specifically, we evaluated the reliability (robustness) of iCSC module by randomly removing a certain percentage of cells and evaluating changes of identified genes in the cell-cell communication networks. The overlapping ratio is measured by the number of shared genes divided by the union of both the output networks. (b) Fold-change effects. we evaluated the reliability by testing different log-fold-change thresholds to pick the important genes. We selected 4 different thresholds 0.04, 0.06, 0.08, and 0.10 respectively, and counted the number of identified genes in the networks. As seen, the number of network nodes decreased with reduced (smaller) fold change thresholds, which is as expected. Also, the networks with higher log-fold-change thresholds are subsets of networks with smaller thresholds. The results indicate the proposed model is reliable. (TIFF) [file pcbi.1011785.s002.tiff]
